# Spleen size evaluation in children: Time to define splenomegaly for pediatric surgeons and pediatricians

**DOI:** 10.1371/journal.pone.0202741

**Published:** 2018-08-23

**Authors:** Gloria Pelizzo, Marinella Guazzotti, Catherine Klersy, Ghassan Nakib, Federico Costanzo, Erika Andreatta, Gabrio Bassotti, Valeria Calcaterra

**Affiliations:** 1 Pediatric Surgery Unit, Children’s Hospital, Istituto Mediterraneo di Eccellenza Pediatrica, Palermo, Italy; 2 Pediatric Surgery Unit, Department of the Mother and Child Health, Fondazione IRCCS Policlinico San Matteo, Pavia, Italy; 3 Biometry & Clinical Epidemiology, Scientific Direction, Fondazione IRCCS Policlinico San Matteo, Pavia, Italy; 4 Department of Paediatric Surgery, Medclinic Middle East, Mediclinic City Hospital, Dubai, United Arab Emirates; 5 Gastroenterology Section, Department of Medicine, University of Perugia Medical School, Perugia, Italy; 6 Pediatric Unit, Department of the Mother and Child Health Fondazione IRCCS Policlinico San Matteo and Department of Internal Medicine University of Pavia, Pavia, Italy; Medizinische Universitat Graz, AUSTRIA

## Abstract

**Background:**

We determined the range of normal spleen dimensions evaluated by ultrasonography (US) in children according to sex and age and the relationship between splenic measurements, auxological data and body proportions, in order to define splenomegaly parameters in support of the surgical mini-invasive approaches in pediatrics.

**Methods:**

We prospectively examined 317 caucasian children of both sexes. The patients were divided into three groups: 0–3 years; 4–10 years; 11–18 years. Sex, weight, height/length, body mass index (BMI), waist circumference and xipho-pubic distance were determined for each child. US spleen evaluation included longitudinal/antero-posterior/transverse diameters, transverse area and volume. Spleen volume/abdominal volume, longitudinal spleen diameter/longitudinal left kidney diameter and longitudinal spleen diameter/xipho-pubic distance ratios were also calculated.

**Results:**

For caucasian subjects, in different age groups spleen volume, transverse area and diameter increased while the spleen/abdominal volume ratio decreased significantly (p<0.001). A significant (p<0.001) decrease in longitudinal spleen diameter/xipho-pubic distance ratio was noted between the 0–3 years group and both 4–10 and 11–18 years group. Age and auxological data, except BMI, showed a high correlation with spleen dimension (r≥0.8).

**Conclusions:**

The current concept of splenomegaly is not applicable in pediatric surgery. A dedicated classification of splenomegaly is needed for children and would improve the safety and feasibility of treatment.

## Introduction

Laparoscopic splenectomy (LS) has gradually become the technique of choice for surgical removal of the spleen also in children [1–6]. Splenic size and its proportions compared to abdominal volume are key factors in determining the feasibility of the laparoscopic approach. Spleen dimensions vary widely according to age. Even though a pathological spleen only becomes palpable once it reaches at least two- three times its normal size [7–8], it may be normally palpable in healthy neonates and children [7–10]. Palpation and percussion are the standard bedside techniques to document spleen size, but are far from being accurate to detect small increases in size [11].

Ultrasonography (US) is an established safe, non-invasive, and reliable method to calculate splenic dimensions [12–15]. In adults, US indicators of moderate splenomegaly include an antero-posterior diameter greater than two thirds of the distance between the anterior and posterior abdominal wall, with a craniocaudal length exceeding the upper limit of 11–14 cm [16]. An interpolar diameter greater than 20 cm should be considered as massive splenomegaly [17].

Unfortunately, there is still no consensus on how to define splenomegaly in pediatric patients. Furthermore, the reference values for B-mode spleen ultrasound reported in the literature are controversial, with differences depending, at least in part, on the ethnic heterogeneity of studied pediatric populations that include subjects with different ethnic backgrounds, country of origin and residence. Racial differences and interfering genetic or infectious factors may influence spleen size [18–24]. Considering that the spleen volume could greatly influence the results of splenectomy, it is mandatory to have normal values for the pediatric population in the geographic area where the surgical approach is performed. An accurate spleen characterization would be useful for planning laparoscopic manoeuvres and determining the specification of dedicated instruments required to perform LS in children.

Aim of this study was to obtain normal percentile values in Caucasian children of both sexes, aged 0–18 years, and to evaluate the relationship between splenic measurements, chronological and auxological data, and body proportions.

## Material and methods

### Subjects

We prospectively examined 317 caucasian healthy children (167 boys, 150 girls) referred to our department between May 2013 and June 2014 for preoperative day surgery workup. A complete auxological evaluation was also obtained before entering the study. The age range was from full-term newborns to 18 years. According to growth spurts, the children were divided into three groups: group 1: 0–3 years (83 subjects, 53M/50F); group 2: 4–10 years (126 subjects, 69M/57F); group 3: 11–18 years (108 subjects, 45M/63F). Sex, weight, height/length, body mass index (BMI), waist circumference and xipho-pubic distance were obtained in each child.

Patients who did not have normal height and weight curves (who were not in the third to 97^th^ percentile according to Cacciari for age≥2 years or to Gairdner’s and Pearson’s for age <2 years) or obese children (BMI>97^th^ according to to Cacciari for age≥2 years) [25–26] were not included in the study. Exclusion criteria also included an history of oncologic and hematologic disorders or infectious causes of splenic enlargement or splenic trauma and accidental discovery of one or more accessory spleens.

### Ethical considerations

After having received information about the nature of the study, the patient’s parents gave written consent for their child’s participation. The study was performed according to the Declaration of Helsinki and with the approval of the Fondazione IRCCS Policlinico San Matteo Review Board.

### Physical examination

This included examination of the participants included evaluation of height, weight, body composition, waist circumference, body mass index (BMI) and xipho-pubic distance.

Below 2 years of age, length was measured by two examiners (one to position the child) with the child supine on a measuring board (infantometer) and weight with an electronic digital scale. In children older than two years, height was measured using a wall mounted Harpenden stadiometer and weight with a standard beam balance scale. Waist circumference was obtained at the midpoint between the lowest rib and the iliac crest. BMI was calculated as body weight (kilograms) divided by body height (meters) squared. Body surface area (BSA) was calculated according to the Mosteller formula: BSA (m^2^) = SQR RT ([Height(cm) x Weight (kg)]/ 3600) [27]. The abdominal volume was computed according to the standard formula: [waist circumference/6.28]^2^* xipho-pubic distance*3.14.

In children older than two years, height, weight, and BMI were classified using Cacciari’s percentiles for the Italian population [25]. In infant, under 2 years of age, length and weight were classified using Gairdner’s and Pearson’s percentiles [26].

### Sonographic evaluation

US spleen evaluation was performed using an Esaote My Lab Twice ultrasound device for image acquisition, with a 3.5–5 MHz convex probe in children above 1 year of age and a up-to-10 MHz linear probe in younger children. All scans were obtained by a single expert sonographer.

All patients were asked to lie down in a supine position or a lateral position when optimal scans were not achievable in the supine position. A water-based medium was applied to both the probe and the body area being scanned to ensure good transmission of the ultrasound beam.

Optimal images for a complete spleen evaluation were obtained through sagittal, transverse, oblique and frontal scans and even though the intercostal space if necessary.

From the obtained images, we measured: longitudinal diameter: between the highest superior-medial and the lower inferior-lateral points of the spleen; antero-posterior diameter: between the anterior and posterior surfaces; transverse diameter: between the hilum and the superior-lateral edge of the spleen; transverse area: delimited by the splenic external margin in the longitudinal scan. Spleen volume was calculated using the prolated ellipsoid formula (L x W x H x 0.523) [28]. We also measured kidney longitudinal diameters with the same technique.

Spleen volume/abdominal volume, longitudinal spleen diameter/longitudinal left kidney diameter, antero-posterior spleen diameter/antero-posterior abdominal diameter and longitudinal spleen diameter/ xipho-pubic distance ratios were also calculated and included in the statistical analyses.

### Statistical analysis

All statistical analyses were performed using STATA 13.1 statistical software (Stata Corporation; College Station, TX, USA). The level of significance was set at the two-tailed P-value <0.05. Data were described with counts, if categorical and with the mean, standard deviation, 25^th^, median, and 75^th^ percentiles if continuous. Both general (for comparison with adults) and pediatric non-parametric reference limits (2.5^th^—97.5^th^ and 3^rd^– 97^th^ percentiles) for spleen measures are reported. Moreover, model-based age specific reference intervals were computed [29], with age modeled as fractional polynomials with the *xriml* user-written routine in Stata [30]. Log transformation of variables was applied before model fitting as needed. The model based 3rd, 10^th^, 50^th^, 90^th^ and 97^th^ percentiles of the (log-transformed) variable were then plotted against age. Spleen measures were compared between age groups with the Kruskall Wallis test. Correlation between spleen measures and clinical characteristics were assessed with the Spearman correlation coefficient (R) and its 95% confidence interval (CI). To assess intra-operator agreement, the Lin’s concordance correlation coefficient (95%CI) [31] and the Bland & Altman Limits of agreement [32] were computed for each splenic diameter.

## Results

**Fig 1:**
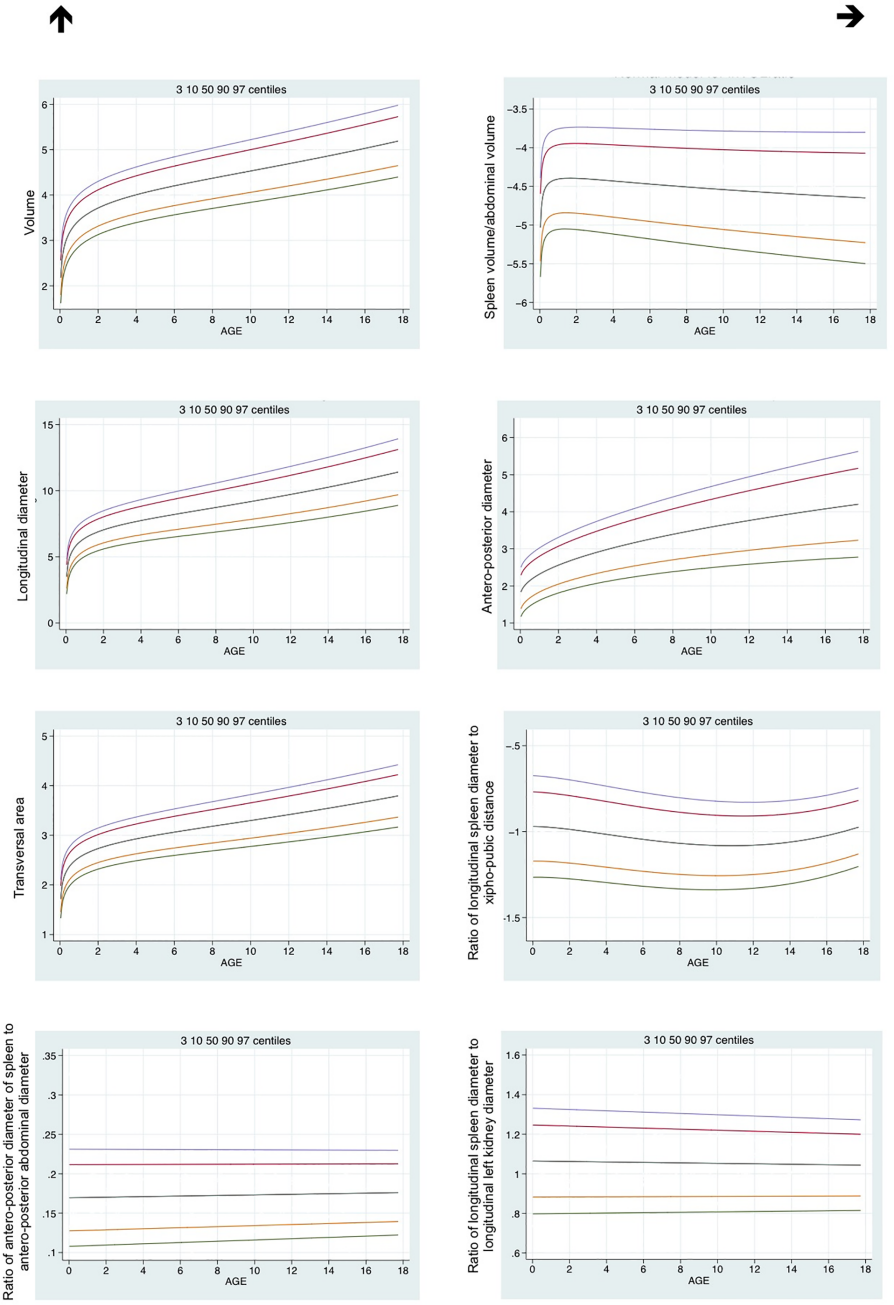
Curves of the model based reference limits (3^th^-97^th^) for the spleen dimensions. Spleen diameters are natural scale; all other measures are log-transformed for the purpose of this analysis.

Clinical characteristics of the children enrolled in the study are reported in Table 1; age groups were well balanced. Spleen dimensions, together with their non-parametric reference limits, are summarized in Table 2, overall and by age group, while the (log transformed) reference limits in relation to age are shown as curves in Fig 1.

**Table 1:** Clinical features of the pediatric subjects.

	Group 1: 0–3 years	Group 1: 4–10 years	Group 3: 11–18 years	Total
Subjects (n)	83	126	108	317
Gender (M/F)	53/30	69/57	45/63	167/150
Age (years)	1.3 (0.9)	6.6 (2.0)	12.6 (1.7)	7.3 (4.7)
Weight (kg)	9.7 (3.5)	25.0 (8.5)	48.0 (12.2)	28.9 (17.6)
Height (m)	0.8 (0.1)	1.2 (0.1)	1.5 (0.1)	1.2 (0.3)
Body surface (m^2^)	0.4 (0.1)	0.9 (0.2)	1.4 (0.2)	1.0 (0.4)
BMI (kg/m^2^)	16.1 (2.1)	16.5 (2.5)	19.9 (3.9)	17.6 (3.4)
Waist circumference (cm)	44.5 (4.7)	57.5 (9.3)	71.3 (9.8)	58.6 (13.5)
Xipho-pubic distance (cm)	16.7 (3.1)	24.1 (3.8)	28.7 (3.0)	23.7 (5.8)

Results expressed as N or mean (SD) as appropriate

**Table 2:** Spleen dimension according to age group. Non parametric reference limits (2.5^th^-97.5^th^) are shown in the last 2 columns.

Variable	mean	SD	median	25th	75th	Kruskall Wallis p-value [& post-hoc comparisons]	2.5th	3rd	97th	97.5th
Volume (cm^3^)						<0.001				
0–18 years	81.0	51.4	70.8	41.2	105.8		13.2	14.9	205.1	221.2
0–3 years	33.1	15.48	31.9	23.5	37.9	0–3 vs 4–10: <0.001	9.3	9.6	67.4	68.3
4–10 years	74.9	30.12	70.2	54.8	90.2	0–3 vs 11–18: <0.001	30.1	30.4	142.3	147.4
11–18 years	125.5	52.14	111.2	90.5	153.0	4–10 vs 11–18: <0.001	42.0	42.6	248.9	264.6
Transverse area (cm^2^)						<0.001				
0–18 years	23.9	11.1	22.7	15.7	30.3		7.1	7.5	47.0	51.1
0–3 years	13.1	4.5	12.8	9.9	15.5	0–3 vs 4–10: <0.001	6.2	6.4	23.8	24.0
4–10 years	22.8	6.0	22.3	18.2	26.8	0–3 vs 11–18: <0.001	12.3	12.5	34.4	36.0
11–18 years	33.6	11.0	32.4	27.8	38.8	4–10 vs 11–18: <0.001	15.1	16.1	55.0	58.5
Longitudinal spleen diameter (cm)						<0.001				
0–18 years	8.4	1.8	8.4	6.9	9.7		4.6	4.7	11.4	11.7
0–3 years	6.2	1.1	6.4	5.7	6.8	0–3 vs 4–10: <0.001	3.5	3.7	8.6	8.7
4–10 years	8.4	1.0	8.3	7.7	9.1	0–3 vs 11–18: <0.001	6.4	6.4	10.6	10.6
11–18 years	9.9	1.2	9.9	9.1	10.8	4–10 vs 11–18: <0.001	7.8	7.9	12.4	12.5
Antero-posterior diameter (cm)						<0.001				
0–18 years	3.2	0.8	3.2	2.6	3.7		1.7	1.7	4.7	4.9
0–3 years	2.4	0.5	2.3	2.1	2.7	0–3 vs 4–10: <0.001	1.4	1.5	3.4	3.4
4–10 years	3.2	0.6	3.2	2.8	3.5	0–3 vs 11–18: <0.001	2.0	2.0	4.4	4.5
11–18 years	3.8	0.7	3.8	3.4	4.2	4–10 vs 11–18: <0.001	2.6	2.7	5.7	6.0
Ratio of spleen volume to abdominal volume (%)						0.17				
0–18 years	1.2	0.5	1.1	0.9	1.4		0.47	0.49	2.21	2.28
0–3 years	1.2	0.5	1.2	0.9	1.4		0.55	0.57	2.52	2.80
4–10 years	1.2	0.5	1.1	0.9	1.5		0.46	0.47	2.36	2.36
11–18 years	1.1	0.4	1.0	0.8	1.4		0.39	0.47	2.02	2.10
Ratio of antero-posterior spleen diameter to antero-posterior abdominal diameter						0.09				
0–18 years	0.17	0.03	0.17	0.15	0.19		0.12	0.11	0.22	0.23
0–3 years	0.17	0.03	0.16	0.15	0.18		0.11	0.11	0.22	0.22
4–10 years	0.18	0.03	0.17	0.15	0.19		0.11	0.10	0.22	0.23
11–18 years	0.17	0.03	0.18	0.15	0.19		0.11	0.10	0.21	0.23
Ratio of longitudinal spleen diameter to xipho-pubic distance (%)						<0.001				
0–18 years	35.8	5.5	35.5	32.6	38.8		25.7	25.8	46.0	47.4
0–3 years	37.8	5.7	37.2	34.3	41.4	0–3 vs 4–10: <0.001	27.1	27.4	51.1	51.9
4–10 years	35.2	5.9	35.2	31.8	37.7	0–3 vs 11–18: <0.001	25.5	25.6	48.9	52.1
11–18 years	35	4.3	34.4	32.1	37.9	4–10 vs 11–18: 0.94	26.2	27.3	45.0	45.2
Ratio of longitudinal spleen diameter to longitudinal left kidney diameter (%)										
0–18 years	105.6	13.4	105.1	96.6	113.6	0.48	83.0	83.6	135.6	136.3
0–3 years	105.6	14.7	105.3	93.7	115.4		77.3	79.5	140.4	145.1
4–10 years	106.6	12.9	107.2	97.8	113.7		85.1	85.8	137.2	139.0
11–18 years	104.4	12.9	103.8	95.8	112.1		82.2	83.2	133.0	134.5

The intra-operator agreement for splenic diameters was excellent for all 3 diameters: longitudinal diameter: rho c = 0.984; 95%CI 0.951–0.995, difference -0.012 (0.301), 95% limits of agreement -0.602–0.579; antero-posterior diameter: rho c = 0.923; 95%CI 0.769–0.975, difference -0.051 (0.295), 95% limits of agreement -0.629–0.528; transverse diameter: rho c = 0.958, 95%CI 0.894–0.984, difference -0.089 (0.332), 95% limits of agreement -0.740–0.562.

Considering that no significant differences in splenic measurements were noted between males and females, statistical analyses were performed by combining the data obtained by both sexes. For all measurements, a significant change according to age group was noted (p<0.001), particularly between the 0–3 and 4–10 years groups. Spleen volume, transverse area and diameters increased and spleen/abdominal volume ratio decreased with age. A decrease in longitudinal spleen diameter/ xipho-pubic distance ratio was noted between the 0–3 years group and the 4–10 and 11–18 years groups. Longitudinal spleen diameter/longitudinal left kidney diameter was greater in the 4–10 years group compared to the 0–3 and 11–18 years group.

Correlations of the spleen dimensions with auxological data and body proportion are reported in Table 3.

**Table 3:** Correlation between spleen dimension and auxological data.

	Weight		Height		Age		Waist circumference		Xipho-pubic distance		BMI		Body surface	
	R (95%CI)	p	R (95%CI)	p	R (95%CI)	p	R (95%CI)	p	R (95%CI)	p	R (95%CI)	p	R (95%CI)	p
Volume (cm^3^)	0.86 (0.83–0.88)	<0.001	0.84 (0.80–0.87)	<0.001	0.82 (0.78–0.85)	<0.001	0.80 (0.76–0.84)	<0.001	0.80 (0.75–0.83)	<0.001	0.53 (0.44–0.60)	<0.001	0.86 (0.83–0.88)	<0.001
Transverse area (cm^2^)	0.85 (0.81–0.87)	<0.001	0.83 (0.80–0.86)	<0.001	0.81 (0.77–0.84)	<0.001	0.80 (0.74–0.83)	<0.001	0.78 (0.74–0.82)	<0.001	0.51 (0.43–0.60)	<0.001	0.84 (0.81–0.87)	<0.001
Longitudinal diameter (cm)	0.86 (0.82–0.88)	<0.001	0.84 (0.80–0.87)	<0.001	0.81 (0.78–0.85)	<0.001	0.80 (0.76–0.84)	<0.001	0.80 (0.76–0.84)	<0.001	0.51 (0.42–0.60)	<0.001	0.86 (0.83–0.88)	<0.001
Antero-posterior diameter (cm)	0.80 (0.75–0.83)	<0.001	0.78 (0.73–0.82)	<0.001	0.77 (0.71–0.81)	<0.001	0.75 (0.70–0.80)	<0.001	0.73 (0.70–0.80)	<0.001	0.47 (0.38–0.55)	<0.001	0.80 (0.75–0.83)	<0.001
Ratio spleen volume/abdominal volume	-0.15 (-0.25–0.33)	<0.001	-0.10 (-0.21–0.00)	<0.001	-0.09 (-0.2–0.01)	0.08	-0.30 (-0.40–0.20)	<0.001	-0.24 (-0.34–0.13)	<0.001	-0.17 (-0.28–0.06)	0.002	-0.14 (-0.02–0.03)	0.01
Ratio of antero-posterior spleen diameter to antero-posterior abdominal diameter	0.05 (-0.06–0.16)	0.37	0.10 (-0.01–0.21)	0.08	0.10 (-0.00–0.21)	0.06	-0.09 (-0.21–0.01)	0.08	0.04 (-0.07–0.15)	0.46	-0.14 (-0.24–0.02)	0.01	0.06 (-0.05–0.17)	0.30
Ratio of longitudinal spleen diameter to xipho-pubic distance	-0.24 (-0.34–0.13)	<0.001	-0.22 (-0.32–0.11)	<0.001	-0.22 (-0.32–0.11)	0.02	-0.28 (-0.38–0.17)	<0.01	-0.46 (-0.54–0.37)	<0.001	-0.08 (-0.20–0.02)	<0.001	-0.24 (-0.34–0.13)	<0.001
Ratio of longitudinal spleen diameter to longitudinal left kidney diameter	0.02 (-0.08–0.13)	0.70	-0.02 (-0.13–0.09)	0.74	-0.03 (-0.14–0.07)	0.5	0.04 (-0.07–0.15)	0.45	0.07 (-0.04–0.18)	0.07	0.11 (0.0–0.2)	0.03	0.101 (-0.09–0.12)	0.01

R: Spearman Correlation Coefficient; CI: confidence limits

Weight, height, body surface, age, waist circumference and xipho-pubic distance were highly correlated with spleen volume, transverse area, longitudinal and antero-posterior diameters. BMI showed the weakest correlation with all organ dimensions measured. No relevant correlation between clinical parameters and spleen volume/abdominal volume, antero-posterior spleen diameter/antero-posterior abdominal diameter, longitudinal spleen diameter/longitudinal left kidney diameter ratios was noted.

**Fig 2:**
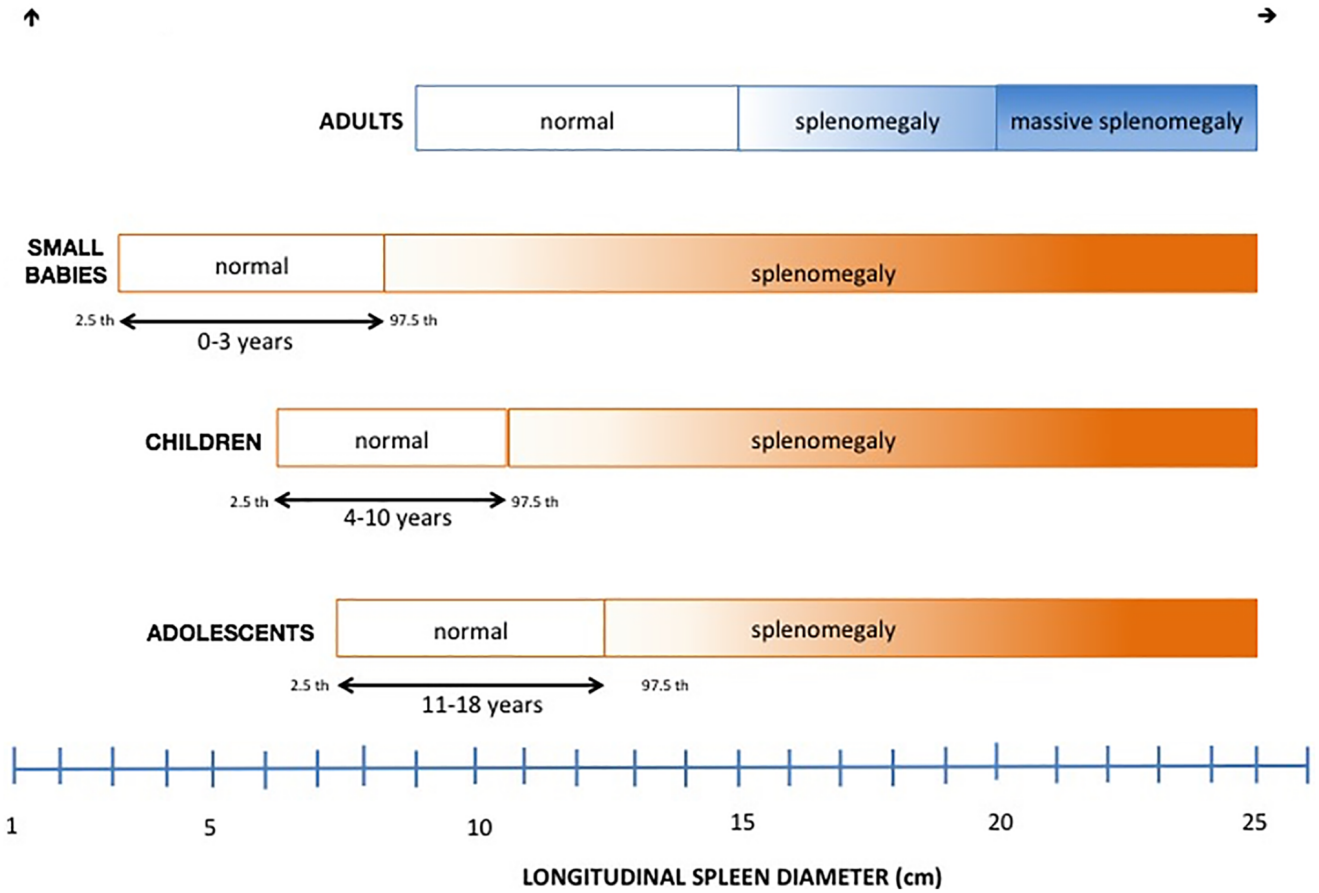
Spleen size in adults [16, 17] and children. Reference limits according to age and our results, are reported as normal values in children.

Specific reference limits for each age from 0 to 18 years are shown as graphical outputs in Fig 2.

## Discussion

We obtained a standard set of normal range splenic dimensions in caucasian children according to three age groups (0–3 years; 4–10 years; 11–18 years). A significant difference between the three age groups was found, particularly between the 0–3 and 4–10 year groups. While all measured spleen dimensions increased with age, though not linearly, their ratios to abdominal volume or kidney diameter appeared constant for the different ages. In addition to spleen length at the hilum, other dimensions were also considered for a more precise spleen size definition. No significant differences in splenic measurements between sexes was noted.

The spleen is easily modified during lifespan due to several physiological and pathological conditions such as growth, infections, hematopoietic disorders, storage diseases, inflammatory conditions as well as splenic trauma. The assessment of organ size is an integral part of the evaluation and treatment of disease, particularly before splenectomy.

Clinical examination is often inaccurate, especially in detecting small increases in size and several studies have documented that palpable cases do not necessarily indicate splenic enlargement [7–9]. Moreover, in some clinical genetic conditions often characterized by the presence of a very large spleen, such as sickle cell disease, Gaucher disease or other storage disorders, the accurate assessment of spleen volume and monitoring of spleen size changes is difficult by spleen palpation. In most individuals, the spleen must be two-three times its normal size before it becomes palpable due to its lateral position and because it is bordered by the ribs, thus making both palpation and percussion difficult [7–8]. Thus, the spleen is usually significantly enlarged before it can be clinically assessed under the costal margin. Moreover, in 15–17% of healthy neonates and in up to 10% of healthy children the spleen can be clinically palpable [7,9,10].

In adults, splenomegaly is defined as a long axis exceeding 15 cm and massive splenomegaly as a long axis exceeding 20 cm [33,34]. Others use spleen weight as an indicative factor [35]. In the SAGES Manual of Strategic Decision Making, simple splenomegaly is defined as a spleen enlarged to more than 50% the average adult organ, and massive splenomegaly as an adult spleen more than 25 cm in length or 900 g in weight [36].

In children, US is routinely used to evaluate the “true” size of abdominal organs because it offers numerous advantages: no radiation exposure, non-invasive technique, examination in real time, three-dimensional and independent of organ function [12–15]. The definition and grading of splenomegaly in children is not standardized. Some authors use the adult criteria, whereas others consider these measurements irrelevant because spleen size is relative to body size [34,37]. The body growth is a differential process, where organs and body segments each enlarge at a specific speed [38,39]. For instance, a child head is relatively larger and heavier than that of an adult; the head and the trunk are relatively large at birth, with progressive lengthening of the limbs throughout development, particularly during puberty. The ratio between the upper and lower body segments decreases with age and is approximately 1.7 at birth, 1.3 at 3 years and 1 after age 7 [40–42]. These particular growth patterns, applied to internal organs, imply that there is a dynamic evolution between each other, and this anthropometric evolution could influence the organ position in the abdominal cavity.

Our data confirm that an increase in organ size is related to growth. Literature data on normal values of pediatric splenic dimensions are not univocal. The dishomogeneous ethnic distribution (and probably related health history) of studied pediatric population, consisting prevalently of Nigerian, Indian, and USA children, may influence the variability of the estimation (also due to “normal” exposure to different pathogens) and may limit the generalizability of the data [18–24].

In addition to literature data [8,10,12–14,43] in this study we provided an unique complete description of splenic measurements including the percentiles in a consistent group of Caucasian children from the same geographic area, from full-term newborns to 18 years, their correlations with auxological data and body proportion and their ratio with abdominal organs.

Contradictory findings on spleen measures and gender association have been previously reported. Some studies indicate a significant difference between males and female in spleen size [19–21]. However, as reported by other authors [8,12,44] in our population no significant differences in splenic dimensions with respect to sex was noted. According to Safak [12], body weight shows the best correlation with spleen dimension; on the contrary, body mass index shows the weakest correlation with all organ measurements [39]. A high correlation between splenic parameters and age, height [18], waist circumference and xipho-pubic distance have also been noted by other authors.

A correlation between organ and abdomen dimensions (volume, transverse area, diameters and ratios between spleen and abdominal dimension) in relation to somatometric parameters would be helpful in defining a “normal “spleen” during growth and could be of paramount importance to define splenomegaly in children [18].

A preoperative spleen size evaluation is crucial for surgeons when planning the feasibility of a LS approach in children, particularly when spleens are greater than 20 cm; these are considered to be “giant” sized spleens, and are difficult to remove laparoscopically in children, due to technical limitations.

Our study showed that the spleen/abdominal volume ratio decreases with age. The spleen volume is relatively larger in infants and toddlers compared to older children. These data confirm the influence of anatomical factors and support the body proportion importance on technical limits during LS in the pediatric age, particularly in young children [45].

LS is a well accepted approach for the treatment of multiple hematologic diseases. Standard surgical steps consist of trocar positioning, initial ligation of the splenic artery to reduce the spleen volume and avoid or reduce any possible bleeding upon spleen extraction. Several different less invasive techniques are available including the single port access splenectomy (SPAS) which reduces the surgery to one small incision; the reduced port access splenectomy (RPAS) which entails the use of fewer and smaller trocars, and the hand-assisted procedure [46,47]. The common retrieval bags in use for tissue morcellation and transumbilical spleen removal measure 12 and 15 cm. In small children, the adaptation of these endo-bags to the small abdominal cavity could represent a real technical challenge. LS for massive splenomegaly in children has generally been found to be associated with a high conversion rate, also including a supplementary suprapubic access to retrieve the spleen and to avoid rupture. Perioperative spleen embolization performed in the operating room prior to laparoscopic splenectomy has been proposed as a safe procedure that can reduce the spleen size and major perioperative complications in children [48]. We feel that, according to our data, when the long axis is less than 12 cm, LS is feasible in all children and adolescents; when the long axis is between 12–15 cm in small infants and children, LS is not safe without suprapubic surgical open access. While in adolescents LS is a safe technique, also without perioperative spleen embolization. When the long axis exceeds 15 cm in small infants and children, LS is not safe; while in adolescents, LS represents a safe approach when perioperative spleen embolization is performed.

Laparoscopic techniques have been shown to offer several advantages over the traditional open technique, in particular: reduced hospital stay, faster return to unrestricted activities, and improved cosmetic results [49]. However, the lack of small instruments and the dedicated technology for the narrow working space in pediatric cases may significantly influence the outcome of LS in children. Adequate pre-operative knowledge of the spleen dimensions and its correlation with auxological parameters would be helpful in defining the most appropriate surgical approach and the choice of dedicated instruments to use. Therefore, it remains to be defined whether the cut-off of 20 cm, the “massive spleen” in adults, is also applicable in children. The European Association for Endoscopic Surgery defines massive splenomegaly in children as a spleen larger than four times normal for age [34]; however, besides normal range, standard deviations must be identified in order to classify the degree of the splenomegaly in pediatrics. Our clinical data indicate that the concept of splenomegaly in adult is not applicable in pediatric cases (Fig 1), and that special consideration should be given to spleen pediatric size in mini invasive surgical treatment of splenomegaly.

## Conclusions

Knowledge of spleen dimensions in relation to clinical data is mandatory to define splenomegaly in children. A dedicated pediatric splenomegaly classification would support the safety and feasibility of mini invasive approaches.

## References

[1] Hassan ME, Al Ali K. Massive splenomegaly in children: laparoscopic versus open splenectomy. JSLS. 2014;18(3). 10.4293/JSLS.2014.00245 PMC4154414 25392624

[2] Kim DJ, Chung JH. Long-term results of laparoscopic splenectomy in pediatric chronic immune thrombocytopenic purpura. Ann Surg Treat Res. 2014;86:314–8. 10.4174/astr.2014.86.6.314 24949323 PMC4062446

[3] Al-Salem AH. Splenectomy for children with thalassemia: total or partial splenectomy, open or laparoscopic splenectomy. J Pediatr Hematol Oncol. 2014 2 26 [Epub ahead of print] 10.1097/MPH.0000000000000121 . 24577545

[4] Vecchio R, Marchese S, Intagliata E. Pediatric laparoscopic splenectomy in a department of general surgery. Updates Surg. 2013;65:337–8. 10.1007/s13304-012-0186-8 23197252

[5] Deng XG, Maharjan A, Tang J, Qiu RL, Wu YH, Zhang J, et al A modified laparoscopic splenectomy for massive splenomegaly in children with hematological disorder: a single institute retrospective clinical research. Pediatr Surg Int 2012;28:1201–9. 10.1007/s00383-012-3215-2 23184263

[6] Corcione F, Pirozzi F, Aragiusto G, Galante F, Sciuto A. Laparoscopic splenectomy: experience of a single center in a series of 300 cases. Surg Endosc. 2012;26:2870–6. 10.1007/s00464-012-2272-x 22580871

[7] French J, Camitta BM. Splenomegaly In: Nelson Textbook of paediatrics. 15^th^ ed Philadelphia, Pa: Saunders 1996;1439.

[8] Megremis SD, Vlachonikolis IG, Tsilimigaki AM. Spleen length in childhood with US: normal values based on age, sex, and somatometric parameters. Radiology. 2004;231:129–34. 10.1148/radiol.2311020963 14990814

[9] Mimouni F, Merlob P, Ashkenazi S, Litmanovitz I, Reisner SH. Palpable spleens in newborn term infants. Clin Pediatr (Phila). 1985;24:197–8. 3978976 10.1177/000992288502400403

[10] Dhingra B, Sharma S, Mishra D, Kumari R, Pandey RM, Aggarwal S. Normal values of liver and spleen size by ultrasonography in Indian children. Indian Pediatr. 2010;47:487–92. 19736366 10.1007/s13312-010-0090-6

[11] Zhang B, Lewis SM. A study of the reliability of clinical palpation of the spleen. Clin Lab Haematol. 1989;11:7–10. 2706905 10.1111/j.1365-2257.1989.tb00168.x

[12] Safak AA, Simsek E, Bahcebasi T. Sonographic assessment of the normal limits and percentile curves of liver, spleen, and kidney dimensions in healthy school-aged children. J Ultrasound Med. 2005;24:1359–64. 16179618 10.7863/jum.2005.24.10.1359

[13] Chiorean L, Zdrenghea M, Badea R. Ultrasonography of the spleen. Pictorial essay. Med Ultrason 2014;16:48–59. 24567925 10.11152/mu.2014.2066.161.lc1mz2

[14] Obikili EN, Anyanwu GE, Onuh AC, Mgbor SO. Sonographic assessment of the normal limits of the spleen in healthy school children in South-East Nigeria. Niger J Clin Pract. 2014;17:484–8. 10.4103/1119-3077.134046 24909474

[15] Benter T, Klühs L, Teichgräber U. Sonography of the spleen. J Ultrasound Med 2011;30:1281–93. 21876100 10.7863/jum.2011.30.9.1281

[16] Robertson F, Leander P, Ekberg O. Radiology of the spleen. Eur Radiol 2001;11:80–95. 10.1007/s003300000528 11194923

[17] Goerg C, Schwerk WB, Goerg K, Havemann K. Sonographic patterns of the affected spleen in malignant lymphoma. J Clin Ultrasound 1990;18:569–74 2170456 10.1002/jcu.1870180708

[18] Megremis S, Alegakis A, Koropouli M. Ultrasonographic spleen dimensions in preterm infants during the first 3 months of life. J Ultrasound Med 200726:329–35. 10.7863/jum.2007.26.3.329 17324982

[19] Ezeofor SN, Obikili EN, Anyanwu GE, Onuh AC, Mgbor SO. Sonographic assessment of the normal limits of the spleen in healthy school children in South-East Nigeria. Niger J Clin Pract 2014; 17: 484–488. 10.4103/1119-3077.134046 24909474

[20] Chow KU, Luxembourg B, Seifried E, Bonig H. Spleen Size Is Significantly Influenced by Body Height and Sex: Establishment of Normal Values for Spleen Size at US with a Cohort of 1200 Healthy Individuals. Radiology 2016; 279:306–313. 10.1148/radiol.2015150887 26509293

[21] Kebede T & Admassie D. Spleen length in childhood with ultrasound normal based on age at Tikur Anbessa Hospital. Ethiop Med J 2009; 47:49–53. 19743780

[22] Eze CU, Agwu KK, Ezeasor DN, Agwuna KK, Aronu AE. Sonographic determination of spleen to left kidney ratio among Igbo school age children of south east Nigeria. Afr Health Sci 2014;14: 246–254. 10.4314/ahs.v14i1.38 26060487 PMC4449051

[23] Eze CU, Agwu KK, Ezeasor DN, Ochie K, Aronu AE, Agwuna KK, Nwadike IU. Sonographic biometry of spleen among school age children in Nsukka, Southeast, Nigeria. Afr Health Sci 2013; 13:384–392. 10.4314/ahs.v13i2.27 24235940 PMC3824484

[24] Al-Imam O, Suleiman A, Khuleifat S. Ultrasound assessment of normal splenic length and spleen-to-kidney ratio in children. East Meditter Health J 2000;6:514–516. 11556047

[25] Cacciari E, Milani S, Balsamo A, Spada E, Bona G, Cavallo L, et al, Cicognani A. Italian cross-sectional growth charts for height, weight and BMI (2 to 20 yr). J Endocrinol Invest. 2006;29:581–93. 10.1007/BF03344156 16957405

[26] Gairdner D & Pearson J. A growth chart for premature and other infants. Arch Dis Child 1971;46:783–787. 5129179 10.1136/adc.46.250.783 PMC1647925

[27] Mosteller RD. Simplified calculation of body-surface area. N Engl J Med 1987; 317: 1098 10.1056/NEJM198710223171717 3657876

[28] Sauerbrei EE, Nguyen KT, Nolan RL. A practical guide to ultrasound in obstetrics and gynecology. New York, Raven Press, 1987, p 15

[29] Wright EM and Royston P. Calculating reference intervals for laboratory measurements. Stat Methods Med Res 1999 8: 93–112 10.1177/096228029900800202 10501648

[30] Wright E, Royston P. Age-specifc reference intervals (normal ranges). Stata Technical Bulletin 1996; 34: 24–34.

[31] Lin LI. A concordance correlation coefficient to evaluate reproducibility. Biometrics. 1989;45:255–68. 2720055

[32] Bland JM, Altman DG. Statistical methods for assessing agreement between two methods of clinical measurement. Lancet. 1986;1:307–10. 2868172

[33] Walsh RM, Brody F & Brown N. Laparoscopic splenectomy for lymphoproliferative disease. Surg Endosc 2004; 18: 272–275. 10.1007/s00464-003-8916-0 14691699

[34] Habermalz B, Sauerland S, Decker G, Delaitre B, Gigot J-F, Leandros E, Lechner K, Rhodes M, Silecchia G, Szold A, Taragona E, Torelli P, Neugebauer E The clinical practise guidelines of the European Association for Endoscopic Surgery (EAES). Surg Endosc 2008;22:821–848. 10.1007/s00464-007-9735-5 18293036

[35] Mahon David & Rhodes Michael. Laparoscopic splenec- tomy: size matters. Ann R Coll Surg Engl 2003;85: 248–251. 12855027 10.1308/003588403766274953 PMC1964390

[36] Skeete D, Swanstrom L, Casaccia M, Sharafuddin M. Splenectomy for massive splenomegaly In: Scott-Conner CEH (ed) The SAGES manual of strategic decision making. Springer, New York, pp 387–403 (2008).

[37] Park A, Heniford BT, Hebra A, Fitzgerald P Pediatric laparoscopic splenectomy. Surg Endosc 2000;14: 527–531. 10890958

[38] Burdi AR. Cephhalometric growth analyses of the human upper face region during the last two trimesters of gestation. Am J Anat. 1969;125:113–22 10.1002/aja.1001250106 5814158

[39] Coquet B, Sandoz B, Savoie PH, Thollon L, Serre T, Brunet C. Anthropometric characterization of spleen in children. Surg Radiol Anat 2010;32:25–30. 10.1007/s00276-009-0535-6 19669612

[40] Roche AF, Mukherjee D, Guo SM. Head circumference growth patterns: birth to 18 years. Hum Biol. 1986;58:893–906. 3557415

[41] Waitzman AA, Posnick JC, Armstrong DC, Pron GE. Craniofacial skeletal measurements based on computed tomography: Part I. Accuracy and reproducibility. Cleft Palate Craniofac J. 1992;29:112–7. 1571344 10.1597/1545-1569_1992_029_0112_csmboc_2.3.co_2

[42] Waitzman AA, Posnick JC, Armstrong DC, Pron GE. Craniofacial skeletal measurements based on computed tomography: Part II. Normal values and growth trends. Cleft Palate Craniofac J. 1992;29:118–28. 1571345 10.1597/1545-1569_1992_029_0118_csmboc_2.3.co_2

[43] Konuş OL, Ozdemir A, Akkaya A, Erbaş G, Celik H, Işik S. Normal liver, spleen, and kidney dimensions in neonates, infants, and children: evaluation with sonography. AJR Am J Roentgenol. 1998;171:1693–98. 10.2214/ajr.171.6.9843315 9843315

[44] Dittrich M, Milde S, Dinkel E, Baumann W, Weitzel D. Sonographic biometry of liver and spleen size in childhood. Pediatr Radiol 1983;13: 206–211. 6888991 10.1007/BF00973157

[45] Tan M, Zheng CX, Wu ZM, Chen GT, Chen LH, Zhao ZX. Laparoscopic splenectomy: the latest technic evaluation.World J Gastroenterol 2003; 9:1086–1089. 10.3748/wjg.v9.i5.1086 12717862 PMC4611378

[46] Monclova JL, Targarona EM, Vidal P, Peraza Y, Garcia F, Otero CR, et al Single incision versus reduced port splenectomy—searching for the best alternative to conventional laparoscopic splenectomy. Surg Endosc 2013; 27: 895–902. 10.1007/s00464-012-2530-y 23052510

[47] Qian D, He Z, Hua J, Gong J, Lin S, Song Z. Hand-assisted versus conventional laparoscopic splenectomy: a systematic review and meta-analysis. ANZ J Surg 2014;84:915–920. 10.1111/ans.12597 24712437

[48] Van Der Veken E, Laureys M, Rodesch G, Steyaert H. Perioperative spleen embolization as a useful tool in laparoscopic splenectomy for simple and massive splenomegaly in children:a prospective study. Surg Endosc 2016; 30:4962–4967. 10.1007/s00464-016-4838-5 26961344

[49] Esposito C, Shaarschmidt K, Settimi A, Montupet P. Experience with laparoscopic splenectomy. J Pediatr Surg 2002;36: 309–11. 10.1053/jpsu.2001.20703 11172422

